# Is resting muscle sympathetic nerve activity related to peripheral or cerebrovascular haemodynamics at high altitude? A retrospective analysis

**DOI:** 10.1113/EP093530

**Published:** 2026-08-19

**Authors:** Ty J. A. MacDonald, Jenna C. McCrone, Christabel Osei‐Boateng, Connor D. Wideman, Samantha L. Tsioros, Joshua C. Tremblay, Alexander Patrician, Connor A. Howe, Geoff B. Coombs, Benjamin S. Stacey, Lydia L. Simpson, Mike Stembridge, Craig D. Steinback, Jonathan P. Moore, Travis D. Gibbons, Ryan L. Hoiland, Francisco Villafuerte, Gilbert Moralez, Philip N. Ainslie, Michael M. Tymko

**Affiliations:** ^1^ Integrative Cerebrovascular and Environmental Physiology SB Laboratory Department of Human Health and Nutritional Sciences College of Biological Science University of Guelph Guelph Ontario Canada; ^2^ Cardiff School of Sport and Health Sciences Cardiff Metropolitan University Cardiff UK; ^3^ Centre for Heart, Lung and Vascular Health, School of Health and Exercise Sciences University of British Columbia – Okanagan Kelowna British Columbia Canada; ^4^ Department of Sport Science University of Innsbruck Innsbruck Austria; ^5^ Institute for Applied Human Physiology Bangor University Bangor UK; ^6^ Neurovascular Research Laboratory University of South Wales Cardiff UK; ^7^ Bristol Medical School University of Bristol Bristol UK; ^8^ Faculty of Kinesiology, Sport, and Recreation University of Alberta Edmonton Alberta Canada; ^9^ Biological Science Northern Arizona University Flagstaff Arizona USA; ^10^ Department of Cellular and Physiological Sciences Faculty of Medicine University of British Columbia Vancouver Canada; ^11^ Laboratorio de Fisiología Comparada/Fisiología del Transporte de Oxígeno Facultad de Ciencias y Filosofía Universidad Peruana Cayetano Heredia Lima Perú; ^12^ Institute for Exercise and Environmental Medicine Texas Health Presbyterian Hospital Dallas and University of Texas Southwestern Medical Center Dallas Texas USA

**Keywords:** blood pressure regulation, brachial artery blood flow, cerebral blood flow, endothelial function, high altitude, sympathetic nerve activity

## Abstract

High‐altitude hypoxia elicits several profound physiological responses, including substantial increases in muscle sympathetic nerve activity (MSNA); however, the association of this heightened MSNA with cardiovascular and cerebrovascular haemodynamics remains unclear in lowlanders and indigenous highlander populations. We hypothesized that at high altitude resting MSNA would be associated with resting cardiovascular and cerebrovascular haemodynamics in lowlanders (*n* = 26), and Sherpa (*n* = 8) and Andean (*n* = 23) indigenous highlanders. Data were retrospectively analysed from two separate high‐altitude expeditions to Nepal (2016; 5050 m) and Peru (2018; 4300 m). We assessed resting MSNA via radial or peroneal microneurography, and measured $S_{pO_{2}}$, heart rate, mean arterial pressure, brachial artery blood flow and endothelial function, and internal carotid and vertebral artery diameter, velocity, and blood flow. Resting MSNA demonstrated limited, population‐specific associations with vascular haemodynamics. Lowlanders showed the most consistent relationships, including positive associations between MSNA and heart rate, vertebral artery diameter and blood flow, and internal carotid artery velocity, while Sherpa and Andean highlanders showed few systemic or cerebrovascular associations with MSNA. No relationship was observed between MSNA and $S_{pO_{2}}$, brachial artery blood flow or endothelial function across populations. Sensitivity analysis confirmed that most directional relationships were robust to the removal of influential participants, although some associations, including the negative relationship between MSNA and brachial artery blood flow in Sherpa, were attenuated. These data highlight that single time‐point, resting MSNA may have limited predictive value for cardiovascular and cerebrovascular haemodynamics at high altitude within these specific populations; however, these data should be interpreted with caution, given the small cross‐sectional sample sizes.

## Abstract (highlights)

- **What is the central question of this study?**
  What is the relationship between resting sympathetic nerve activity and cardiovascular and cerebrovascular haemodynamics in lowlanders and indigenous highlanders.
- **What is the main finding and its importance?**
  These data provide new insight into the limited contribution of resting peripheral sympathetic nerve activity on cardiovascular and cerebrovascular haemodynamics at high‐altitude, with lowlanders exhibiting more consistent relationships than Indigenous Sherpa or Andean highlanders.

## 1 INTRODUCTION

Adaptation to high‐altitude environments above ∼4000 m entails several physiological adaptations that enable the body to survive in severe hypoxic conditions. Enhanced muscle sympathetic nerve activity (MSNA) is a characteristic of chronic exposure to high altitude, causing sustained increases by 2‐ to 3‐fold (Hansen & Sander, 2003; Tymko, Lawley et al., 2020; Tymko et al., 2023). Despite research highlighting the prevalence of increased MSNA at high altitude, its impact on cardiovascular and cerebrovascular haemodynamics remains limited. Although this neural response likely plays an important adaptive role, helping counteract hypoxia‐induced vasodilation to maintain mean arterial pressure (MAP), it may also restrict the vasculature's ability to appropriately dilate under certain conditions (Lewis et al., 2014; Tymko, Tremblay, Steinback et al., 2017; Tremblay, Howe et al., 2018, Tremblay, Hoiland et al., 2018; Tymko, Lawley et al., 2020). This suggests a functional link between elevated sympathetic nerve activity (SNA) and altered vascular regulation at altitude.

SNA plays a central role in regulating beat‐to‐beat blood pressure and heart rate (HR). While MSNA is tightly linked to HR due to its cardiac gating, its relationship with resting MAP is less straightforward and influenced by compensatory adjustments in cardiac output and peripheral resistance (Hart et al., 2012). For example, in healthy young adults, there is no relationship between resting MSNA and MAP, potentially due to compensatory mechanisms between cardiac output and total peripheral resistance (Hart et al., 2012). At high altitude, despite MSNA being 2–3 times higher (Tymko et al., 2023), there is only a modest increase in MAP, indicating that similar to sea‐level, a minimal relationship between MSNA and MAP likely exists in young and healthy individuals at high altitude. However, while MAP may be relatively insensitive to resting MSNA in young individuals, emerging evidence shows that acute changes in SNA can directly influence peripheral artery endothelial function at low altitude (Atkinson et al., 2015; Dyson et al., 2006; Hijmering et al., 2002; Thijssen et al., 2014) and high altitude (Tymko, Lawley et al., 2020). This raises the possibility that chronically elevated MSNA may have important effects on resting cardiovascular haemodynamics.

In contrast, the influence of chronic sympathoexcitation on cerebrovascular haemodynamics is even less clear. The cerebrovasculature is densely innervated by sympathetic nerve fibres, but the significance of their input remains controversial, with evidence that both supports and refutes the notion that SNA contributes to cerebral blood flow (CBF) regulation (Brassard et al., 2017; Koep et al., 2022). Previous high‐altitude work indicates that despite sympathoexcitation, sympathetic blockade does not alter resting CBF, indicating that SNA may play a limited role in maintaining cerebral haemodynamics during chronic high‐altitude exposure (Ainslie et al., 2012). However, no study has directly examined whether resting peripheral MSNA is associated with cardiovascular and cerebrovascular haemodynamics at high altitude, nor whether these relationships differ between acclimatizing lowlanders and indigenous Sherpa and Andean highlanders.

High‐altitude indigenous populations, such as the Sherpa and Andean highlanders, have evolved distinct physiological adaptations from living in their respective high‐altitude environments for several millennia (Beall, 2006, 2007). For example, there is evidence that Sherpa highlanders have preserved peripheral and cerebrovascular function at high altitude when compared to lowlanders in order to maintain oxygen delivery (Tremblay, Hoiland *et al.*, 2018; Tymko, Lawley et al., 2020). In contrast, Andean highlanders maintain oxygen delivery via pronounced increases in haemoglobin concentration and haematocrit (Beall, 2006, 2007; Villafuerte & Corante, 2016), but also exhibit susceptibility to chronic mountain sickness, impaired vascular function and heightened SNA (Tremblay, Coombs *et al.*, 2019; Tymko, Lawley et al., 2020).

Therefore, the purpose of the present study was to examine the associations between resting MSNA (burst frequency and amplitude) and cardiovascular and cerebrovascular haemodynamics in lowlanders, Sherpa and Andean highlanders at high altitude. This investigation used retrospective data from two previous high‐altitude expeditions to Nepal and Peru (Tymko, Hoiland et al., 2020; Willie et al., 2018) to evaluate how these relationships may differ across populations with distinct levels of acclimatization and long‐term adaptation. We hypothesized that at high altitude, resting MSNA would be positively associated with HR and blood pressure, but negatively associated with peripheral and cerebral vascular blood flow. Furthermore, these associations would be more pronounced in lowlanders acclimatizing to high altitude, compared to highlanders who have chronically adapted to their respective environments, indicating potential phenotypic differences between lowlanders and highlanders.

## 2 METHODS

### 2.1 Ethical approval

The data presented were collected in accordance with the standards set by the latest revision of the *Declaration of Helsinki*, except for registration in a database. The studies were approved by the University of British Columbia (H17‐02687 and H18‐01404), Universidad Peruana Cayetano Heredia (no. 101686), and Nepal Health Research Council (no. 227/2016). As detailed elsewhere, all participants provided informed written consent after each study was thoroughly explained in their native language (Tymko, Hoiland et al., 2020; Willie et al., 2018).

### 2.2 Participants

Data were collected during two high‐altitude research expeditions conducted in the Khumbu Valley, Nepal (5050 m) and Cerro de Pasco, Peru (4300 m). All physiological measurements for both lowlander and highlander participants were obtained at these high‐altitude field sites (>4000 m). Lowlander participants were born and currently reside at low‐altitude (<1500 m). In contrast, all highlander participants and both of their parents were born and have permanently resided at high altitude (>3000 m). The presented data (neural and vascular) were collected after at least 1 week of sojourn at high altitude; however, these data were collected on different days, during separate experiments, while at high altitude. Participants were screened for hypertension and had no history of cardiovascular and cerebrovascular disease. Participants were tested a minimum of 6 h post‐meal, 12 h post‐caffeine and 24 h post‐exercise.

### 2.3 Cardiovascular measurements

Measurements of HR and blood pressure were recorded using three separate approaches across all data: (1) using an automated blood pressure cuff that outputted both HR and blood pressure (systolic, diastolic and MAP; Omron, Kyoto, Japan), or (2) continuous recordings, with electrocardiogram electrodes placed in lead II configuration (Bioamp, ML132, ADInstruments, Colorado Springs, CO, USA), and blood pressure via finger photoplethysmography (Finometer Pro, Finapres Medical Systems, Amsterdam, Netherlands). The automated blood pressure cuff data were averaged over two to three stable measurements, and the continuous recordings were averaged over a 1‐min period during the participants baseline period, or (3) using intra‐arterial blood pressure measurements, the VAMP blood sampling kit (VAMP Adult, Edwards Lifescience, Irvine, CA, USA) was connected to a blood pressure amplifier (ADInstruments, FE117) and was calibrated (i.e., zeroed to room air) at the level of the fourth intercostal space for continuous blood pressure recording.

### 2.4 Systemic vascular function

Participants were equipped with a cuff located on their left forearm, fixed at the level of their heart. Brachial artery image acquisition was obtained using a 15‐MHz multifrequency linear array probe attached to a high‐resolution ultrasound machine (15L4 transducer, Terason uSmart 3300, Burlington, MA, USA). Following optimal image acquisition, and 1 min of baseline recordings, the forearm was occluded by inflating the cuff to 220 mmHg for 5 min. Recordings of diameter and velocity resumed 30 s before cuff deflation and continuously for 3 min thereafter (Thijssen et al., 2011). Brachial artery data presented in this manuscript have been published previously (Tymko, Tremblay, Steinback et al., 2017; Tremblay, Howe et al., 2018, Tremblay, Hoiland et al., 2018, Tremblay, Grewal et al., 2019, Tremblay, Hoiland et al., 2019, Tremblay, Coombs et al., 2019); however, the current investigation answers a novel research question through retrospective analysis related to the relationship between basal MSNA and brachial artery vascular function. Ultrasound recordings were continuously captured for offline analysis.

Brachial artery data were assessed using automated edge‐detection and wall‐tracking software, which integrates synchronous diameter and velocity measurements at 30 Hz to minimize investigator bias (Woodman et al., 2001). Flow‐mediated dilation (FMD) was calculated as the percent increase in diameter from baseline to peak following cuff release, with diameters automatically extracted from continuous data.

### 2.5 Cerebrovascular ultrasonography

Simultaneous B‐mode and Doppler ultrasonography were performed to assess continuous diameter and blood flow velocity recordings of the internal carotid artery (ICA) and vertebral artery (VA) using a 15‐MHz linear array probe attached to a high‐resolution ultrasound machine (15L4 transducer, Terason uSmart 3300). Ultrasound techniques were based on previously established guidelines (Thomas et al., 2015). Insonation angle remained unchanged at 60 degrees throughout the entire protocol. Ultrasound recordings were screen captured and saved for offline analysis. A minimum of 10 consecutive cardiac cycles were analysed at least 2 cm away from the carotid bifurcation and averaged for measurements of CBF.

### 2.6 MSNA

A tungsten microelectrode (50 mm length, 200 µm diameter) was percutaneously inserted into the peroneal nerve (laying supine) or ultrasound guided into the radial nerve (lying semi‐recumbent); recording has been shown to be similar between these sites (Rea & Wallin, 1989). A reference electrode was placed subcutaneously 1–3 cm from the recording site. Suitable nerve sites were located via manual manipulation until a pulse‐synchronous burst pattern was identified. MSNA signals were confirmed by the absence of paresthesias and an increase during voluntary apnoea but not to loud noise. The signal was amplified 1000× via a preamplifier and 100× by a variable‐gain amplifier, band‐pass filtered (700–2000 Hz), sampled at 10,000 Hz, and stored for offline analysis (LabChart V7.1, ADInstruments). The MSNA data included here have been reported previously (Berthelsen et al., 2020; Busch et al., 2020; Hansen et al., 2022; Simpson et al., 2019, 2020; Tymko, Tremblay, Steinback et al., 2017, Tymko, Lawley et al., 2020). Resting MSNA was averaged over at least 5 min of breathing and expressed as burst frequency and mean burst amplitude.

### 2.7 Statistics

All statistical analyses were performed using GraphPad Prism (v10.2.3; GraphPad Software, Boston, MA, USA) and R (RStudio v2026.01.2+418). Differences in participant demographic characteristics were assessed using a one‐way analysis of variance (ANOVA), with Tukey's *post hoc* test applied when significant interactions were observed. To examine associations between MSNA and cardiovascular and cerebrovascular variables, separate linear regression models were constructed in R (lm) using either MSNA burst frequency or burst amplitude as continuous predictor variables. To assess whether these relationships differed across populations (lowlanders, Sherpa and Andeans), additional models included population as a categorical fixed effect along with an interaction term between MSNA and population group, thereby allowing comparison of regression slopes between groups. To facilitate pairwise comparisons between all three populations, each interaction model was run three times with each population serving as the reference group in turn. All regression analyses were performed using ordinary least squares methods and no random effects were included. 95% confidence intervals are reported for all parameter estimates. Data are presented as means ± standard deviation. Given the small sample sizes reported, statistical significance was not defined solely by a threshold of *P* < 0.05. Accordingly, relationships that approached this threshold were interpreted cautiously, with consideration of both *P*‐values and confidence intervals from the raw data. The strength of associations was evaluated using adjusted coefficients of determination (adjusted *R* ^2^), and 95% confidence intervals. To evaluate model sensitivity, influential observations in the regression models were assessed using Cook's distance, with values >1 considered indicative of influential points. Analysis with removal of influential variables (Cook's *D* > 1, or ±2 SD from the mean) is interpreted in the Results and Discussion.

## 3 RESULTS

### 3.1 Participants and baseline data

The study participants that had measurements of MSNA, cardiovascular and cerebrovascular haemodynamics from the two high‐altitude expeditions to Nepal (Willie et al., 2018) and Peru (Tymko, Hoiland et al., 2020) consisted of 26 lowlanders (2 females), 8 Sherpa (all male) and 23 Andean highlanders (all male). The participant demographics and resting data are presented in Table 1. Of the Andean highlanders, eight presented with excessive erythrocytosis ([Hb] > 21 g/dL). Lowlander and Sherpa highlander participants were not different in age (27.6 ± 5.8 years and 29.4 ± 12.7 years old, respectively), but Andean highlanders were older (43.7 ± 12.7 years; *P* < 0.01). Lowlanders were taller (176.8 ± 5.3 cm; *P* < 0.01) compared to both Sherpa and Andean highlanders (165.8 ± 7.6 cm and 160.3 ± 5.1 cm, respectively). Lowlanders weighed more than Sherpa highlanders (73.8 ± 9.1 kg vs 60.7 ± 11.6 kg; *P* = 0.02), but not compared to Andean highlanders (69.1 ± 11.6 kg). Lowlander body mass index was lower (23.6 ± 2.9 kg/m^2^) compared to Andean highlanders (26.8 ± 4.3 kg/m^2^; *P* < 0.02). Sherpa highlanders had a slightly lower peripheral oxygen saturation ($S_{pO_{2}}$) (80.6 ± 4.2%; *P* = 0.02) compared to Andean highlanders (84.8 ± 3.5%), but was not different from lowlanders (83.0 ± 3.9%; *P* = 0.26). Andean highlanders had higher haemoglobin concentration (20.5 ± 2.1 g/dL; *P* < 0.001), compared to both lowlanders and Sherpa (15.9 ± 1.4 g/dL and 15.7 ± 1.0 g/dL). Andean highlanders also had higher haematocrit (62.1 ± 7.0%), compared to both lowlanders and Sherpa (46.3 ±  3.4% and 46.1 ±  2.7%, both *P* < 0.001).

**TABLE 1:** Lowlander and highlander participant demographics.

	LL	Sherpa	Andean
Age (years)	27.6 ± 5.8	29.4 ± 12.7	43.7 ± 12.7
BMI (kg/m^2^)	23.6 ± 2.9^‡^	23.6 ± 5.1	26.8 ± 4.3
$S_{pO_{2}}$ (%)	83.0 ± 3.9	80.6 ± 4.2^‡^	84.8 ± 3.5
[Hb]	15.9 ± 1.4^‡^	15.7 ± 0.1^‡^	20.5 ± 2.1
Hct (%)	46.3 ± 3.4^‡^	46.1 ± 2.7^‡^	62.1 ± 7.0
HR (bpm)	69.1 ± 16.6	72.4 ± 6.9	72.8 ± 8.2
MAP (mmHg)	90.8 ± 11.1	83.3 ± 9.3	90.0 ± 10.6
BA blood flow (mL/min)	27.5 ± 7.6^†^	69.7 ± 41.8	32.5 ± 20.2^†^
BA FMD (mm)	0.195 ± 0.1	0.274 ± 0.08	0.268 ± 0.11
BA FMD (%)	4.7 ± 3.0	6.5 ± 2.2	6.6 ± 3.0
ICA diameter (mm)	4.95 ± 0.42	4.75 ± 0.42^‡^	5.33 ± 0.69
ICA velocity (cm/s)	42.3 ± 6.7^‡^	38.8 ± 4.8^‡^	32.2 ± 5.2
ICA blood flow (mL/min)	249.4 ± 51.0	208.6 ± 43.3	214.5 ± 77.3
VA diameter (mm)	3.87 ± 0.57	3.62 ± 0.53	3.79 ± 0.53
VA velocity (cm/s)	25.2 ± 7.8	28.0 ± 5.0	16.37 ± 7.6^*†^
VA blood flow (mL/min)	94.4 ± 44.1	89.3 ± 32.5	60.2 ± 37.3^*^
MSNA burst frequency (bursts/min)	31.0 ± 12.4	22.1 ± 10.6^*‡^	34.1 ± 13.6
MSNA burst amplitude (a.u.)	44.82 ± 9.5	51.5 ± 7.7^*‡^	44.2 ± 7.4

*Note*: Values are represented by mean ± standard deviation. Statistical significance (*P* < 0.05): * vs Lowlander (LL), † vs Sherpa, ‡ vs Andean.

Abbreviations: BA, brachial artery; BMI indicates body mass index; DBP, diastolic blood pressure; [Hb], haemoglobin concentration; Hct, haematocrit; ICA, Internal carotid artery; MAP, mean arterial pressure; SBP, Systolic blood pressure; $S_{pO_{2}}$, peripheral oxygen saturation; VA, vertebral artery.

Lowlanders had a burst frequency range of 13.8–75.4 bursts/min, Sherpa of 9.0–42.0 bursts/min and Andeans of 18.0–61.2 bursts/min. Burst amplitude ranges were 25.9–58.9 in lowlanders, 34.0–58.0 in Sherpa and 28.6–53.2 in Andean highlanders. A consistent inverse relationship was observed between burst frequency and burst amplitude in Lowlanders (*r* = −0.265 [95% CI: −0.592, 0.136], *P* = 0.191), Sherpa (*r* = −0.335 [95% CI: −0.841, 0.484], *P* = 0.417), and Andeans (*r* = −0.308 [95% CI: −0.721, 0.266], *P* = 0.284), though within‐group correlations were not statistically significant.

### 3.2 Resting MSNA and haemodynamics within lowlanders and highlanders

Across individual populations, the relationships between MSNA burst frequency and burst amplitude, and vascular haemodynamics are presented in Table 2. Among lowlanders, MSNA burst frequency was positively associated with HR (95% CI: 0.250, 1.009; *P* = 0.002) and ICA blood velocity (95% CI: 0.011, 0.395; *P* = 0.03), while all other relationships were non‐significant. MSNA burst amplitude in lowlanders showed a negative association with HR (95% CI: −1.203, −0.100; *P* = 0.022), a weak negative association with MAP (95% CI: −0.901, 0.035; *P* = 0.069), a positive association with VA diameter (95% CI: 0.001, 0.006; *P* = 0.008) and blood flow (95% CI: 0.295, 3.871; *P* = 0.024), and a negative relationship with ICA blood velocity (95% CI: −0.542, 0.000; *P* = 0.050). No other vascular or oxygenation measures were related to MSNA amplitude in lowlanders. In Sherpa, MSNA burst frequency showed minimal associations to BA blood flow (95% CI: −0.417, 2.922; *P* = 0.137), and a weak negative association to ICA blood flow (95% CI: −6.967, 1.668; *P* = 0.222). However, MSNA burst amplitude was strongly associated with BA blood flow (95% CI: −6.728 −2.814; *P* < 0.001), although this relationship was largely driven by one influential variable, and when removed became obsolete. MSNA burst amplitude also appeared to have a weak correlation to VA blood flow (95% CI: −6.720, 1.033; *P* = 0.145) and velocity (95% CI: −1.227, 0.225; *P* = 0.169). In Andeans, MSNA burst frequency appeared to have a correlation to MAP, although the confidence interval includes zero as a plausible outcome (95% CI: −0.054, 0.618; *P* = 0.098). MSNA burst amplitude only appeared to be correlated to BA blood flow (95% CI: −0.423, 2.380; *P* = 0.163).

**TABLE 2:** Resting MSNA and haemodynamics within lowlanders and highlanders.

	MSNA burst frequency			MSNA burst amplitude		
	LL	Sherpa	Andean	LL	Sherpa	Andean
$S_{pO_{2}}$ (%)	0.013 [−0.113, 0.138], *P* = 0.843	0.095 [−0.181, 0.372], *P* = 0.492	−0.031 [−0.153, 0.092], *P* = 0.619	−0.064 [−0.225, 0.096], *P* = 0.423	−0.045 [−0.419, 0.329], *P* = 0.810	−0.094 [−0.379, 0.192], *P* = 0.512
HR (bpm)	0.629 [0.250, 1.009], ***P* = 0.002**	0.372 [−0.465, 1.208], *P* = 0.377	0.082 [−0.288, 0.452], *P* = 0.658	−0.652 [−1.203, 0.100], ***P* = 0.022**	0.03 [−1.253, 1.314], *P* = 0.962	0.143 [−0.835, 1.122], *P* = 0.769
MAP (mmHg)	0.142 [−0.203, 0.486], *P* = 0.413	0.064 [−0.695, 0.824], *P* = 0.866	0.282 [−0.054, 0.618], *P* = 0.098	−0.433 [−0.901, 0.035], *P* = 0.069	0.493 [−0.596, 1.581], *P* = 0.366	−0.123 [−0.835, 1.122], *P* = 0.767
BA blood flow (mL/min)	−0.072 [−1.524, 1.380], *P* = 0.920	1.253 [−0.417, 2.922], *P* = 0.137	−0.268 [−1.090, 0.554], *P* = 0.512	−0.87 [−3.222, 1.482], *P* = 0.454	−4.77 [−6.728, −2.814], ***P *< 0.001**	0.978 [−0.423, 2.380], *P* = 0.163
BA FMD (mm)	<0.001 [−0.005, 0.006], *P* = 0.987	0.003 [−0.003, 0.009], *P* = 0.269	<0.001 [−0.002, 0.004], *P* = 0.662	<0.001 [−0.004, 0.005], *P* = 0.727	−0.003 [−0.010, 0.004], *P* = 0.536	<0.001 [−0.006, 0.004], *P* = 0.818
BA FMD (%)	−0.022 [−0.205, 0.160], *P* = 0.805	0.098 [−0.121, 0.317], *P* = 0.371	−0.008 [−0.115, 0.100], *P* = 0.889	−0.046 [−0.238, 0.147], 0.632	−0.008 [−0.307, 0.291], *P* = 0.956	−0.008 [−0.222, 0.207], *P* = 0.943
ICA diameter (mm)	<0.001 [−0.003, 0.001], *P* = 0.268	−0.002 [−0.006, 0.002], *P* = 0.248	<0.001 [−0.001, 0.003], *P* = 0.378	<0.001 [−0.002, 0.002], *P* = 0.806	0.003 [−0.003, 0.008], *P* = 0.333	<−0.001 [−0.006, 0.005], *P* = 0.87
ICA velocity (cm/s)	0.203 [0.011, 0.395], ***P* = 0.039**	−0.17 [−0.586, 0.246], *P* = 0.412	0.011 [−0.221, 0.242], *P* = 0.927	−0.271 [−0.542, 0.000], *P* = 0.05	0.07 [−0.514, 0.655], *P* = 0.808	0.07 [−0.541, 0.682], *P* = 0.816
ICA blood flow (mL/min)	−0.035 [−2.008, 1.939], *P* = 0.972	−2.65 [−6.967, 1.668], *P* = 0.222	0.453 [−1.947,2.852], *P* = 0.705	−0.894 [−3.841, 2.054], *P* = 0.541	2.237 [−4.143, 8.616], *P* = 0.480	−0.158 [−6.828, 6.513], *P* = 0.962
VA diameter (mm)	<−0.001 [−0.003, 0.001], *P* = 0.471	<−0.001 [−0.005, 0.004], *P* = 0.791	<−0.001 [−0.003, 0.002], *P* = 0.599	0.003 [0.001, 0.006], ***P* = 0.008**	−0.002 [−0.008, 0.003], *P* = 0.370	<0.001 [−0.006, 0.005], *P* = 0.871
VA velocity (cm/s)	0.079 [−0.172, 0.330], *P* = 0.529	−0.041 [−0.586, 0.505], *P* = 0.881	−0.021 [−0.324, 0.282], *P* = 0.889	0.199 [−0.138, 0.535], *P* = 0.238	−0.501 [−1.227, 0.225], *P* = 0.169	−0.093 [−1.118, 0.931], *P* = 0.804
VA blood flow (mL/min)	−0.17 [−1.544, 1.204], *P* = 0.804	−0.332 [−3.340, 2.676], *P* = 0.824	−0.276 [−1.948, 1.396], *P* = 0.74	2.083 [0.295, 3.871], ***P* = 0.024**	−2.844 [−6.720, 1.033], *P* = 0.145	−0.116 [−4.169, 3.937.], *P* = 0.954

*Note*: Linear regression analyses performed separately within lowlander (LL), Sherpa and Andean groups for each cardiovascular and cerebrovascular variable. For each outcome, MSNA burst frequency and MSNA burst amplitude were entered as independent predictors in separate models. Values represent the regression coefficient (β), 95% confidence interval (CI) and *P*‐value for each within‐group association. Values shown in bold achieved statistical significance (*P* < 0.05).

### 3.3 Comparison of resting MSNA and haemodynamics between lowlanders and highlanders

**FIGURE 1:**
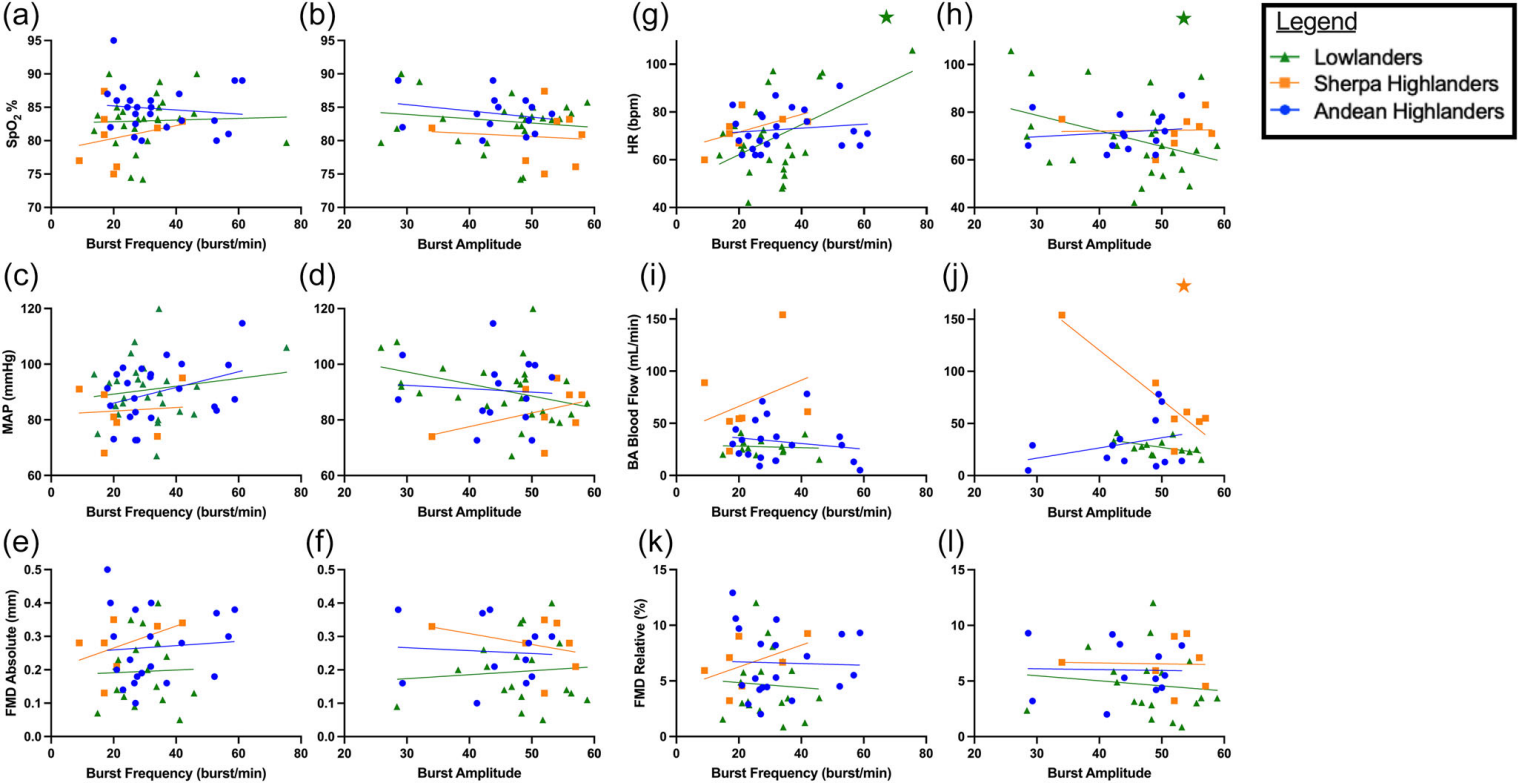
Relationships between MSNA burst frequency and MSNA burst amplitude and cardiovascular variables in lowlanders (green), Sherpa (orange) and Andean (blue) participants. For each group, a separate linear model was used to assess within‐group associations; coloured lines represent regression slopes. No between‐group statistical comparisons are displayed in the figure, and results reflect within‐group simple linear relationships only. Coloured star indicates that individual group regression achieved significance (P < 0.05).

**FIGURE 2:**
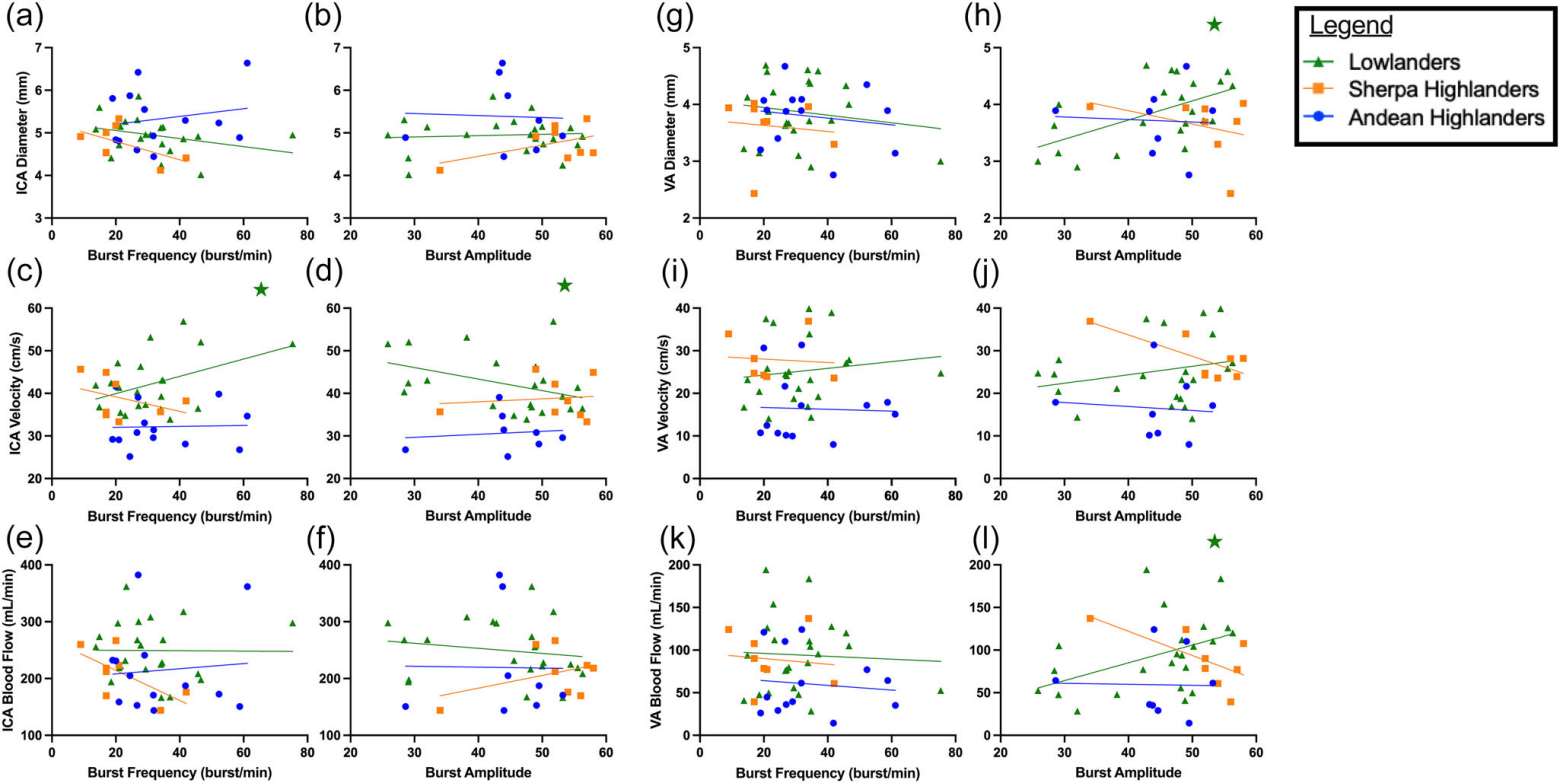
Relationships between MSNA burst frequency and MSNA amplitude and cerebrovascular variables in lowlanders (green), Sherpa (orange) and Andean (blue) participants. For each group, a separate linear model was used to assess within‐group associations; coloured lines represent regression slopes. No between‐group statistical comparisons are displayed in the figure, and results reflect within‐group simple linear relationships only. Coloured star indicates that individual group regression achieved significance (P < 0.05).

Across group comparisons examining whether the strength or direction of MSNA and vascular relationships differed between lowlanders, Sherpa and Andeans, showing that for most variables, the associations were not statistically different across populations (Table 3; Figures 1 and 2). For both MSNA burst frequency and MSNA burst amplitude models, $S_{pO_{2}}$, MAP, BA FMD (absolute and percent), ICA and VA diameter, velocity, and flow, all demonstrated non‐significant MSNA interaction relationships (*P* > 0.150 for all comparisons), indicating that the slopes relating MSNA to these variables did not differ between high‐altitude populations.

**TABLE 3:** Comparison of resting MSNA and haemodynamics between lowlanders and highlanders.

	MSNA burst frequency			MSNA burst amplitude		
	LL vs Sherpa	LL vs Andean	Sherpa vs Andean	LL vs Sherpa	LL vs Andean	Sherpa vs Andean
$S_{pO_{2}}$ (%)	−0.221, 0.386; *P* = 0.586	−0.218, 0.132; *P* = 0.625	−0.428, 0.176; *P* = 0.407	−0.388, 0.427; *P* = 0.923	−0.356, 0.298; *P* = 0.859	−0.519, 0.422; *P* = 0.836
HR (bpm)	−1.176, 0.660; *P* = 0.576	−1.077, −0.018; ***P* = 0.043**	−1.204, 0.625; *P* = 0.528	−0.715, 2.079; *P* = 0.330	−0.328, 1.918; *P* = 0.161	−1.501, 1.727; *P* = 0.888
MAP (mmHg)	−0.911, 0.756; *P* = 0.853	−0.341, 0.622; *P* = 0.560	−0.612, 1.049; *P* = 0.600	−0.259, 2.111; *P* = 0.122	−0.642, 1.263; *P* = 0.515	−1.984, 0.753; *P* = 0.369
BA blood flow (mL/min)	−0.888, 3.537; *P* = 0.232	−1.864, 1.472; *P* = 0.813	−3.381, 0.341; *P* = 0.106	−6.961, −0.842; ***P* = 0.014**	−0.890, 4.586; *P* = 0.177	3.343, 8.156; ***P* ≤ 0.001**
BA FMD (mm)	−0.004, 0.011; *P* = 0.562	−0.005, 0.006; *P* = 0.835	−0.009, 0.004; *P* = 0.534	−0.012, 0.005; *P* = 0.479	−0.008, 0.006; *P* = 0.686	−0.006, 0.011; *P* = 0.711
BA FMD (%)	−0.165, 0.405; *P* = 0.399	−0.197, 0.227; *P* = 0.950	−0.349, 0.138; *P* = 0.388	−0.318, 0.393; *P* = 0.832	−0.250, 0.326; *P* = 0.789	−0.367, 0.369; *P* = 0.997
ICA diameter (mm)	−0.005, 0.003; *P* = 0.556	−0.001, 0.005; *P* = 0.169	−0.001, 0.007; *P* = 0.153	−0.004, 0.008; *P* = 0.437	−0.007, 0.006; *P* = 0.804	−0.011, 0.005; *P* = 0.431
ICA velocity (cm/s)	−0.831, 0.085; *P* = 0.107	−0.493, 0.108; *P* = 0.202	−0.295, 0.657; *P* = 0.446	−0.303, 0.986; *P* = 0.288	−0.327, 1.010; *P* = 0.305	−0.845, 0.846; *P* = 0.999
ICA blood flow (mL/min)	−7.362, 2.132; *P* = 0.272	−2.620, 3.594; *P* = 0.753	−1.837, 8.042; *P* = 0.211	−3.897, 10.158; *P* = 0.371	−6.556, 8.029; *P* = 0.838	−11.624, 6.836; *P* = 0.601
VA diameter (mm)	−0.004, 0.005; *P* = 0.951	−0.003, 0.003; *P* = 0.956	−0.005, 0.005; *P* = 0.981	−0.012, 0.000; *P* = 0.053	−0.010, 0.002; *P* = 0.206	−0.006, 0.010; *P* = 0.613
VA velocity (cm/s)	−0.720, 0.481; *P* = 0.689	−0.494, 0.294; *P* = 0.61	−0.604, 0.643; *P* = 0.95	−1.500, 0.100; *P* = 0.084	−1.122, 0.539; *P* = 0.479	−0.643, 1.458; *P* = 0.435
VA blood flow (mL/min)	−3.469, 3.145; *P* = 0.921	−2.270, 2.058; *P* = 0.922	−3.385, 3.498; *P* = 0.974	−9.196, −0.658; ***P* = 0.025**	−6.629, 2.231; *P* = 0.320	−2.881, 8.336; *P* = 0.330

*Note*: Pairwise between group comparisons for the associations between MSNA burst frequency and MSNA burst amplitude and each cardiovascular and cerebrovascular variable. Values represent 95% confidence intervals and *P*‐values derived from linear regression models testing group differences in the relationship (i.e., differences in regression slopes) between lowlanders (LL), Sherpa and Andean participants. Values in bold achieved statistical significance (*P* < 0.05).

For MSNA burst frequency, only the association between MSNA and HR differed between lowlanders and Andeans (95% CI: −1.077, −0.018; *P* = 0.043), primarily driven by a positive association in lowlanders that was absent in Andeans (see Table 3). No other comparisons were apparent. For MSNA burst amplitude, BA blood flow displayed significant group differences, with lowlanders differing from Sherpa (95% CI: −6.961, −0.842; *P* = 0.014) and Sherpa differing from Andeans (95% CI: 3.343, 8.156; *P* < 0.001), although this difference disappeared with the removal of an influential variable in the Sherpa population. Additionally, VA diameter showed a trending difference between lowlanders and Sherpa (95% CI: −0.012, 0.000; *P* = 0.053), and VA blood flow differed between lowlanders and Sherpa (95% CI: −9.196, −0.658; *P* = 0.025), consistent with population‐specific variability in MSNA and VA blood flow coupling.

### 3.4 Sensitivity analysis

To assess for any potential influence of individual observations of linear regression estimates, Cook's distance was calculated for all models. Using a threshold of Cook's *D* > 1.0, several observations were identified as potentially influential. In the burst frequency models, two participants had high influence (*D* = 1.52 and *D* = 1.01) on the brachial artery blood flow model. In the burst amplitude models, one participant had high influence (*D* > 1.14) for HR, brachial artery blood flow and ICA blood flow models. One participant had high influence (*D* = 4.04) on the burst amplitude and ICA blood flow model. The influential observations were included in the primary analysis to preserve the integrity of the full data set.

The sensitivity analysis revealed few changes in directionality of the slope coefficients, and confidence intervals of the associations between MSNA and vascular variables in lowlanders and highlander populations. Two key relationships in Sherpa were altered in the sensitivity analysis. With the removal of two Sherpa, MSNA burst frequency and BA blood flow differed in direction (β = −0.225 [95% CI: −1.684, 1.235]), and the correlation between MSNA burst amplitude and BA blood flow disappeared (95% CI: −8.355, 3.386; *P* = 0.390). All correlations were deemed insignificant in the Sherpa population after removal of influential participants; however, due to the small sample sizes typically associated with high‐altitude field research (e.g., *n* = 8 Sherpa, *n* = 23 Andean), all participants were included in the primary dataset, and all interpretations of these associations should be viewed as exploratory and may not be fully representative of their respective populations as a whole.

## 4 DISCUSSION

The present study examined the extent to which resting MSNA relates to vascular haemodynamics in acclimatizing lowlanders and indigenous Sherpa and Andean highlanders, living at high‐altitude. Contrary to our initial hypothesis, our data suggest that there were only limited associations between MSNA and vascular haemodynamics, with no consistent relationships observed within individual populations. Furthermore, MSNA was not associated with $S_{pO_{2}}$, which is thought to be the driver of elevated MSNA at high altitude. While lowlanders exhibited several unexpected associations between MSNA and cerebrovascular variables, neither Sherpa nor Andean participants demonstrated similar patterns, which could reflect phenotypic differences in how chronic hypoxia and lifelong altitude exposure shape neural–vascular interactions. Given the rarity of these study populations, emphasis should be placed on the magnitude and direction of observed relationships along with the associated confidence intervals, rather than statistical significance alone. The present findings provide physiological insight into these populations and further work is necessary to understand the haemodynamic regulation of these unique models of long‐term human adaptation to hypoxia.

### 4.1 Resting MSNA and haemodynamics within lowlanders and highlanders

When examined within each population by itself, some associations between MSNA and haemodynamics emerged. Ranges of burst frequency and burst amplitude were reported, with burst amplitude having comparable distributions, while the burst frequency ranges differed substantially between populations, with lowlanders having a notably wider range of frequencies recorded (13.75–75.35 bursts/min).

Lowlanders exhibited a clear positive relationship between HR and MSNA burst frequency (95% CI: 0.250, 1.009; *P* = 0.002), alongside a negative relationship between HR and burst amplitude (95% CI: −1.203, 0.100; *P* = 0.022). This is likely because individuals with high burst frequency rates had lower burst amplitudes at high altitude (*r* = −0.349; *P* = 0.02). Lowlanders also had a borderline negative association between burst amplitude and MAP (95% CI: −0.901, 0.035; *P* = 0.069). This relationship was predominately negative in direction but remained uncertain as confidence intervals included zero, suggesting a potential inverse relationship between sympathetic burst amplitude and MAP. High‐altitude exposure is often associated with a blunted vascular sympathetic transduction (Berthelsen et al., 2020), and our results indicate that, to some extent, resting sympathetic drive may have some association to resting arterial blood pressure in high‐altitude hypoxia, although this is in contrast with data at sea‐level that indicate that resting MSNA is not associated with blood pressure (Hart et al., 2012).

In Sherpa, the only systemic association with MSNA appeared to be with BA blood flow, which had some association to MSNA burst frequency (95% CI: −0.417, 2.922; *P* = 0.137) and amplitude (95% CI: −6.728, −2.814; *P* < 0.001), although these relationships disappeared with the removal of influential participants. No other systemic haemodynamic variable showed clear relationships with MSNA, as the associations were weak and imprecise, with confidence intervals spanning zero and substantial uncertainty. Overall, Sherpa highlanders exhibited lower resting MSNA burst frequency and higher MSNA burst amplitude compared with both lowlanders and Andean highlanders, aligning with previously published work (Simpson et al., 2020).

In Andeans, burst frequency showed a possible positive association with MAP (95% CI: −0.054, 0.618; *P* = 0.098) as the confidence interval suggests a potential, but uncertain, positive relationship including zero as a plausible outcome. There was also a small positive trend between burst amplitude and BA blood flow (95% CI: −0.423, 2.380; *P* = 0.163) although this confidence interval included zero as an outcome. No other measure of systemic haemodynamics had a meaningful association, with confidence intervals lacking consistent directionality. The disparity in MSNA–peripheral vascular coupling between populations provides further support that phenotypic differences exist between lowlanders and highlanders.

In contrast to our hypothesis, resting MSNA was not correlated with BA blood flow or endothelial function. We expected an inverse relationship, given that hypoxia‐induced sympathetic activation, although likely necessary for maintaining systemic arterial pressure, can also impose vascular restraint. Instead, the absence of any association suggests that resting MSNA may not provide ample physiological insight into peripheral artery vascular health at rest at high altitude. Previous work shows that acute elevations in SNA impair endothelial function and blunt vasomotor capacity at sea‐level (Atkinson et al., 2015; Dyson et al., 2006; Hijmering et al., 2002; Thijssen et al., 2014), but not at high altitude (Tymko, Tremblay, Hansen et al., 2017, Tymko, Tremblay, Steinback et al., 2017). Together, these findings are consistent with the possibility that chronic high‐altitude exposure may blunt the sympathetically mediated vascular restraint typically observed at sea‐level, potentially through structural or functional adaptations in the peripheral vasculature.

Cerebrovascular associations were most evident in lowlanders. ICA velocity was positively associated with MSNA burst frequency (95% CI: 0.011, 0.395; *P* = 0.039) as well as a possible negative association with burst amplitude (95% CI: −0.542, 0.000; *P* = 0.05). VA diameter and blood flow were positively related to burst amplitude (95% CI: 0.001, 0.006; *P* = 0.008; and 95% CI: 0.295, 3.871; *P* = 0.024), showing relationships in the anterior and posterior cerebral haemodynamics. Neither Sherpa nor Andeans exhibited a clear relationship between CBF and MSNA, and the reason for this is currently unclear. There were some small directional trends in Sherpa, exhibiting a possible negative trend between MSNA burst frequency and ICA blood flow (95% CI: −6.967, 1.668; *P* = 0.222), and MSNA burst amplitude and VA velocity (95% CI: −1.227, 0.225; *P* = 0.169) and blood flow (95% CI: −6.720, 1.022; *P* = 0.145) although all of these associations include zero as a possible outcome, and therefore a level of uncertainty remains when interpreting these data.

The role of SNA on regulating CBF has been controversial in humans (reviewed in: Brassard et al., 2017; Koep et al., 2022), but there is some evidence that SNA at rest influences CBF (Tymko, Fraser et al., 2020), and strong physiological stressors can increase cerebral noradrenaline spillover, which has the potential to signal changes in cerebrovascular function (Tymko et al., 2025), but this area of research requires further attention. In the context of sympathetic control of CBF at high altitude, there is only one study to investigate this to date (Ainslie et al., 2012). The authors reported that pharmacological blockade of SNA in healthy humans impaired dynamic cerebral autoregulation, suggesting a role for the sympathetic nervous system in regulating CBF. However, it is important to note that cerebrovascular reactivity to carbon dioxide was unchanged in this study, and the pharmacological agents used produced substantial reductions in resting blood pressure, making the interpretation of these findings challenging. The data from the current study slightly challenge some of the findings by Ainslie et al. (2012), since the current data indicate that a modest relationship might exist between resting MSNA and cerebrovascular haemodynamics. However, these relationships largely disappear when removing influential data points (see ‘Sensitivity analysis’ section below). Future work should aim to confirm this finding to rule out potential type 1 statistical error. The relationship between MSNA and cerebrovascular haemodynamics was detected exclusively in lowlanders. Whether these lowlander‐specific relationships reflect adaptive mechanisms to support perfusion in response to high‐altitude acclimatization, or represent a maladaptive response that contributes to altitude‐related illness remains unclear, but they highlight a potential population‐specific difference in sympathetic cerebrovascular control.

### 4.2 Comparison of resting MSNA and haemodynamics between lowlanders and highlanders

Few cross‐population differences were present in these data. MSNA burst frequency had significantly different relationships (*P* = 0.043) from HR between lowlanders and Andeans, which was largely driven by a relationship between MSNA and HR present in lowlanders, but was absent in Andeans. The association between MSNA burst amplitude and BA blood flow was significantly different between lowlanders and Sherpa (*P* = 0.014), and between Sherpa and Andeans (*P* < 0.001). These findings align with the data presented in Table 3, which show a strong inverse relationship between MSNA and BA blood flow uniquely in Sherpa. However, this finding should be interpreted with caution due to the potential influence of influential participants on the overall model. These data indicate that sympathetic burst size may exert differential vascular effects across population. In the cerebrovasculature, VA blood flow showed a significantly different relationship with MSNA burst amplitude between lowlanders and Sherpa (*P* = 0.025), in that high MSNA burst amplitude was associated with higher levels of VA blood flow, suggesting that the posterior cerebrovasculature of lowlanders may be more sensitive to tonic SNA compared to Sherpa highlanders, although this relationship was absent in the ICA.

### 4.3 Sensitivity analysis

Analysis of the dataset with influential participants removed (determined via Cook's Distance > 1, or MSNA burst frequency or amplitude >± 2 SD from the mean within‐population) led to changes in the certainty of several associations, but the overall pattern and directionality of most relationships were preserved. Notably, the positive association between lowlander MSNA burst frequency and HR, the positive association between Andean MSNA burst frequency and MAP, and the positive associations between lowlander MSNA burst amplitude and VA diameter and VA blood flow remained directionally consistent following removal of influential participants. In contrast, the negative association between Sherpa MSNA burst amplitude and BA blood flow was substantially attenuated. In several of the previously observed cerebrovascular relationships in lowlanders, directionality of the relationship remained similar; however, the confidence of the associations became less certain, and many included zero as a plausible outcome of the relationship. A small number of additional associations emerged following removal of influential participants, including inverse relationships between Andean MSNA burst amplitude and ICA blood flow and ICA diameter.

### 4.4 Methodological limitations

Although the data were collected under controlled conditions at high altitude, variations in sympathetic tone and vascular responsiveness could still occur due to external factors such as sleep quality, hydration, diet, fitness levels and stress, none of which can be entirely standardized in field studies at high altitude. Another major limitation is that these data are cross sectional. A more meaningful comparison of these relationships would be to assess the change in MSNA against the change in vascular haemodynamics between low altitude and high altitude. Although some of these data exist in the lowlanders, these data do not exist in the highlanders who were only tested at one time point at high altitude. The small sample size for Sherpa limits confidence in population‐specific interpretation; the data set is also predominately male, and further inclusive studies are needed. Between‐group differences in height, weight and age could also influence arterial health and blood flow and potentially confound the association between MSNA and peripheral and cerebral haemodynamics. However, due to the relatively small sample size, we did not do any further analysis as doing so would reduce the statistical power.

Heart rate was either obtained from three‐lead electrocardiogram, or calculated via the continuous blood pressure waveforms, and blood pressure was measured via finger plethysmography (i.e., Finometer) or an intra‐arterial blood pressure transducer. It is possible that different collection techniques would add variability to our data sets; however, this variability is likely minimal given all data collection was completed under normal resting conditions. Another limitation is that the measures may not have been made on the same day at high altitude, and therefore day‐to‐day variability in these measures could have contributed to the lack of associations observed between MSNA and the vasculature. Furthermore, this study assumes that MSNA provides a proxy for sympathetic activity to the organ systems assessed. It is important to acknowledge, however, that peripheral MSNA does not necessarily reflect cardiac or cerebral sympathetic drive (Macefield, 2013). Further, due to the large number of linear regressions performed on the limited dataset, the potential for type 1 error was large. To combat this, a sensitivity analysis was performed with removal of potentially influential participants. This analysis was performed to test the strength of these associations, and to investigate if there were any substantial differences (i.e. directionality switch of the slope coefficient) in the observed associations of the primary analysis.

### 4.5 Conclusion

This study demonstrates that single‐time‐point, resting MSNA shows limited and largely population‐specific associations with vascular haemodynamics at high altitude. Associations with cerebrovascular variables were most evident in lowlanders, while Sherpa and Andean highlanders displayed few consistent relationships, potentially reflecting phenotypic differences shaped by lifelong hypoxic exposure. Sensitivity analysis indicated that most directional patterns were preserved after removing influential participants, although several associations lost statistical precision. Given the investigative nature of the presented data, modest sample sizes, and heterogeneity between populations, these cross‐sectional findings should be interpreted with caution and viewed as exploratory rather than confirmatory. Collectively, these results provide preliminary insight into the relationship between sympathetic activity and vascular haemodynamics at high altitude across populations, and highlight the need for larger, mechanistic studies to clarify blood flow regulation during chronic hypoxic exposure.

## AUTHOR CONTRIBUTIONS

Michael M. Tymko and Ty J. A. MacDonald were responsible for the concept of the manuscript. All authors contributed to the analysis, interpretation of the data, along with drafting the article or critically revising it for important intellectual content. All authors have read and approved the final version of this manuscript and agree to be accountable for all aspects of the work in ensuring that questions related to the accuracy or integrity of any part of the work are appropriately investigated and resolved. All persons designated as authors qualify for authorship, and all those who qualify for authorship are listed.

## CONFLICT OF INTEREST

The authors declare no conflicts of interest.

## GENERATIVE AI STATEMENT

Generative AI (Claude Sonnet 4.6; Anthropic) was used to assist in the development and editing of the statistical code. All code was verified and executed by the authors in Rstudio (v2026.01.2+418). The authors confirm all other content including drafting and revisions of this manuscript was conceived, written, and approved solely by the authors.

## Data Availability

The datasets used and/or analysed during the current study are available from the corresponding author on reasonable request.
